# Health care access, affordability, and satisfaction following enrollment in Medicare Advantage

**DOI:** 10.1093/haschl/qxag202

**Published:** 2026-08-11

**Authors:** Andrew S Oseran, Bassel M Shanab, Archana Tale, Yunzhe Qian, Rishi K Wadhera

**Affiliations:** Richard A. and Susan F. Smith Center for Outcomes Research, Beth Israel Deaconess Medical Center, Boston, MA 02215, USA; Division of Cardiology, Beth Israel Deaconess Medical Center, Boston, MA 02215, USA; Department of Health Policy & Management, Harvard T.H. Chan School of Public Health, Boston, MA 02215, USA; Richard A. and Susan F. Smith Center for Outcomes Research, Beth Israel Deaconess Medical Center, Boston, MA 02215, USA; Richard A. and Susan F. Smith Center for Outcomes Research, Beth Israel Deaconess Medical Center, Boston, MA 02215, USA; Richard A. and Susan F. Smith Center for Outcomes Research, Beth Israel Deaconess Medical Center, Boston, MA 02215, USA; Division of Cardiology, Beth Israel Deaconess Medical Center, Boston, MA 02215, USA

**Keywords:** Medicare Advantage, patient-reported outcomes, access, affordability, satisfaction

## Abstract

**Introduction:**

Medicare Advantage (MA) enrollment is growing rapidly, yet less is known about how these plans impact health care access, affordability, and satisfaction for adults newly entering Medicare.

**Methods:**

Event study, difference-in-differences analysis comparing longitudinal changes in patient-reported outcomes among adults newly enrolled in MA (vs traditional Medicare [TM]) in the Health and Retirement Study (2010-2022).

**Results:**

There were 4247 adults who newly enrolled in Medicare between 65 and 67 years of age (34.2% MA; 65.8% TM). Following Medicare enrollment, health care access improved similarly in MA and TM, with no differential change between groups. However, MA enrollees experienced a greater reduction in the proportion of individuals reporting trouble affording medical care (difference-in-differences [DiD] estimate −5.3pp [95% CI, −9.7 to −0.9]). Additionally, the percentage of adults satisfied with their care increased for both groups; however, these gains were larger among MA enrollees and increased over time (DiD +13.3pp [95% CI, 7.9-18.6]).

**Conclusion:**

In this national, longitudinal study of adults entering Medicare, we found that enrollment in MA was associated with greater improvements in the affordability of care and higher satisfaction compared with enrollment in TM, without meaningful differences in health care access.

## Introduction

More than 34 million adults are now enrolled in privately administered Medicare Advantage (MA) plans in the United States.^1^ However, MA remains controversial, attracting widespread attention from clinicians, policymakers, and industry leaders. Supporters contend that MA promotes competition and innovation within the Medicare program, ultimately benefiting enrollees. Critics, however, argue that MA is an expensive alternative that has yet to deliver meaningful improvements in outcomes. The rapid growth of MA over the past decade has therefore fueled efforts to better understand how well these private plans serve beneficiaries.^2^

Many previous studies comparing care quality and outcomes in MA compared with traditional Medicare (TM) have been cross-sectional and susceptible to selection bias because beneficiaries do not choose insurance plans at random. For example, individuals with greater medical needs or serious health events are more likely to enroll in TM, making it difficult to determine whether observed differences in outcomes reflect plan design or underlying population characteristics.^3-6^ One way to mitigate this limitation is through longitudinal analyses that leverage the natural experiment occurring at age 65, when most adults transition into a Medicare plan due to age-based eligibility rather than a health shock or life event. One study has employed this design but relied on claims from a single commercial insurer—limiting generalizability.^7^ Moreover, because studies comparing MA and TM frequently use claims or other administrative data sets, patient-centered outcomes like health care access, affordability, and satisfaction, which represent important dimensions of plan quality from patients' perspective, remain underexplored.

To address these gaps, in this study, we used data from the Health and Retirement Study—a national, longitudinal survey—to examine 3 key questions. First, is enrollment in MA associated with changes in health care access relative to enrollment in TM? Second, are there changes in the affordability of care following MA enrollment? And third, are there differences in satisfaction with care between new MA and TM enrollees?

## Methods

### Data sources and study population

The Health and Retirement Study (HRS) is a longitudinal, national panel study of US adults over the age of 50, sponsored by the National Institute on Aging and conducted by the University of Michigan.^8^ Interviews are conducted every 2 years via telephone or in-person, and detailed information on demographics, health insurance, health status and use, retirement, income, and wealth is collected. For this study, we used data from the 2010 through 2022 survey waves. The 2020 wave incorporated methodological adaptations due to the COVID-19 pandemic.^9^

Our study cohort included adults who newly enrolled in Medicare between 65 and 67 years of age. The purpose of this age restriction was to isolate adults who enrolled in Medicare primarily due to age-related changes in insurance eligibility (rather than another life or medical event).^7^ We divided our cohort based on whether individuals enrolled in MA or TM. Individuals had to have data from at least one survey cycle before and after Medicare enrollment to be included, and we excluded anyone who was enrolled in Medicare prior to the age of 65 (Appendix Figure S1). No beneficiaries in our cohort switched between MA and TM during the follow-up period. Baseline demographic (age, sex, race/ethnicity, insurance type of origin, education, employment, census division, household income) and clinical (hypertension, hyperlipidemia, diabetes, heart disease, stroke, cancer, lung disease, psychiatric disease) characteristics were collected based on survey responses in the wave immediately prior to Medicare transition (survey cycle −1).

### Outcomes

Our primary outcomes fell into 3 distinct domains: (1) health care access, (2) affordability of health care, and (3) satisfaction. Health care access was based on whether an individual reported (1) having no usual source of care and (2) trouble finding a health care provider. Affordability of care was based on whether an individual reported (1) skipping medical care due to cost and (2) taking less medication than prescribed due to cost. Finally, satisfaction was based on whether an individual reported being “very satisfied” or “somewhat satisfied” with the quality, cost, and convenience of their health care. Because the HRS survey is administered every 2 years, all outcomes represent an average over a 2-year period.

### Statistical analysis

We first evaluated baseline sociodemographic and clinical characteristics of beneficiaries who newly enrolled in MA and TM. We then used a quasi-experimental difference-in-differences design with an event study framework to assess changes in outcomes following Medicare enrollment, comparing beneficiaries who newly enrolled in MA to those who newly enrolled in TM.^10-13^ This study design allowed us to account for the staggered timing of Medicare enrollment (intervention) across calendar years as well as the possibility that treatment effects might vary over time. Medicare enrollment was considered time 0, and we evaluated outcomes for up to 3 survey cycles (or 6 calendar years) before (survey −3, −2, −1) and after (survey +1, +2, +3) enrollment.

We used the Callaway-Sant’Anna doubly robust estimator, which combines outcome regression and propensity score weighting, to estimate dynamic treatment effects for each survey cycle relative to Medicare enrollment, with survey cycle −1 serving as the reference.^13^ We fit linear regression models that included an indicator variable for Medicare plan type (MA or TM), interaction terms between plan type and survey cycle relative to enrollment, and calendar-year fixed effects to account for national secular trends. For each survey cycle, the coefficient of the interaction term represents the difference-in-differences estimate of the effect of enrolling in MA on an outcome relative to the effect of enrolling in TM. For example, in the regression model for trouble finding a provider, the interaction coefficient for relative year +1 reflects how the change in trouble finding a provider among MA enrollees (relative to their pre-enrollment level at survey cycle −1) compares with the corresponding change for TM enrollees. All models adjusted for the sociodemographic and clinical characteristics in Table 1. The parallel trends assumption was assessed both visually and by statistical testing for all outcomes.

**Table 1:** Baseline characteristics of adults who newly enrolled in Medicare Advantage and traditional Medicare between 65 and 67 years of age.

	Medicare Advantage *N* (%)	Traditional Medicare *N* (%)
**Unweighted sample**	1451	2796
**Mean age (years)**	63.6	63.5
**Female sex**	881 (60.7)	1619 (57.9)
**Race and ethnicity**		
NH White	806 (55.6)	1857 (66.4)
NH Black	317 (21.9)	468 (16.7)
Hispanic	277 (19.1)	380 (13.6)
Other	51 (3.5)	90 (3.2)
**Baseline insurance**		
Private	1066 (73.5)	2035 (72.8)
Medicaid	117 (8.1)	159 (5.7)
VA/military	33 (2.3)	206 (7.4)
Uninsured	231 (15.9)	387 (13.8)
**Educational attainment**		
Not high-school graduate	174 (12.0)	293 (10.5)
High-school graduate or GED	727 (50.1)	1320 (47.2)
Some college	145 (10.0)	263 (9.4)
College graduate	405 (27.9)	920 (32.9)
**Annual household income**		
<25^th^ percentile	470 (32.4)	715 (25.6)
25^th^ to 50^th^ percentile	378 (26.1)	695 (24.9)
50^th^ to 75^th^ percentile	245 (16.9)	504 (18.0)
>75^th^ percentile	356 (24.5)	880 (31.5)
**Census division**		
New England	43 (3.0)	73 (2.7)
Mid Atlantic	143 (10.1)	298 (10.9)
East North Central	226 (15.9)	437 (15.9)
West North Central	55 (3.9)	205 (7.5)
South Atlantic	314 (22.1)	670 (24.4)
East South Central	82 (5.8)	191 (7.0)
West South Central	161 (11.3)	320 (11.7)
Mountain	105 (7.4)	212 (7.7)
Pacific	291 (20.5)	337 (12.3)
**Chronic conditions**		
Hypertension	839 (57.8)	1612 (57.7)
Diabetes	358 (24.7)	701 (25.1)
Hyperlipidemia	737 (50.8)	1427 (51.0)
Heart disease	237 (16.3)	457 (16.3)
Stroke	66 (4.6)	122 (4.4)
Cancer (non-skin)	142 (9.8)	344 (12.3)
Chronic lung disease	111 (7.7)	209 (7.5)
Psychiatric/neurologic disease	424 (29.2)	747 (26.7)
Current smoker	163 (11.2)	347 (12.4)

Missingness was <0.5% for all variables.

Abbreviations: NH, non-hispanic; VA, veterans affairs.

To assess the influence of propensity score weighting and covariate adjustment on our doubly robust estimates, we conducted a sensitivity analysis comparing estimates from unadjusted outcome regressions, covariate-adjusted outcome regressions, and inverse probability weighted (IPW)-only regressions. As a secondary analysis, we repeated our primary analyses stratified by baseline insurance type (private, Medicaid, uninsured) to examine whether findings differed by plan of origin. Analyses restricted to VA/military insurance coverage were not feasible due to insufficient sample sizes.

All analyses were conducted in R (version 4.4.2) using the Callaway-Sant’Anna estimator provided in the *did* R package.^14,15^ We report 95% confidence intervals based on wild cluster bootstrapping with 1000 iterations. Statistical significance was assessed at an α level of 0.05. This study used publicly available, de-identified data from HRS, which does not constitute human subjects research under US federal regulations. Thus, institutional review board approval was not required.

## Results

The study population included 4247 adults who newly enrolled in Medicare between 65 and 67 years of age. There were 1451 (34.2%) new MA enrollees and 2796 (65.8%) new TM enrollees. Baseline characteristics of the study population are presented in Table 1. Adults who newly enrolled in MA were less likely to be White (55.6% vs 66.4%) and had lower household income (32.4% vs 25.6% less than 25^th^ percentile). The distribution of baseline insurance prior to Medicare enrollment and the burden of co-morbidities were similar between groups.

### Health care access

**Figure 1:**
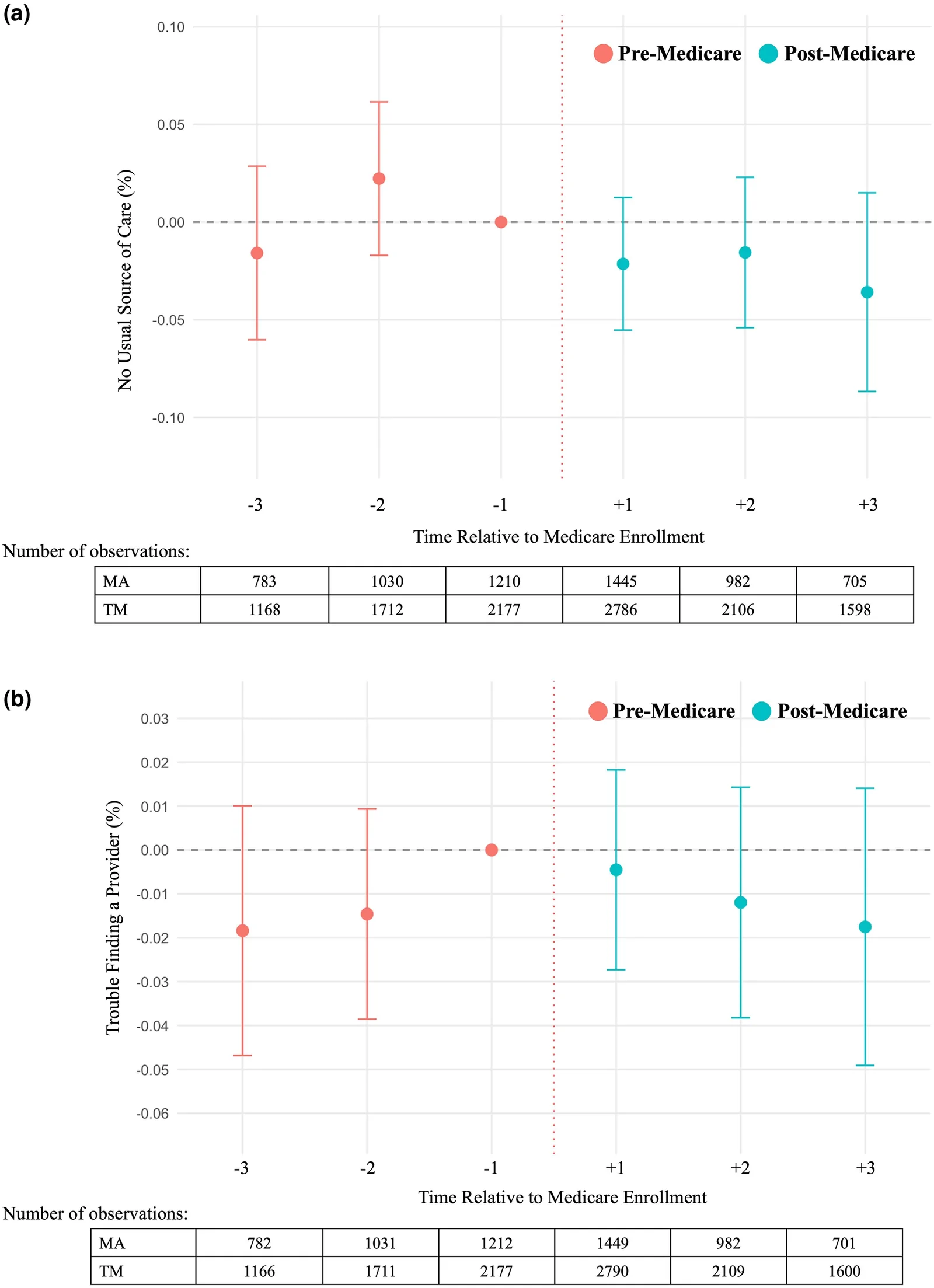
Differential change in health care access before and after Medicare enrollment. The figures describe the differential change in outcomes of interest by survey cycle before and after Medicare enrollment (vertical dashed line). Participants in the Health and Retirement Study are interviewed every 2 years; therefore, survey cycle +3 is approximately 6 years after Medicare enrollment. The red dots describe differential change in outcomes before Medicare enrollment, and the blue dots describe differential change in outcomes after Medicare enrollment. The whisker is 95% confidence interval. Each outcome represents the average over a 2-year period. The *P*-values for interaction in the pre-period were 0.86 (usual source of care) and 0.18 (trouble accessing care), supporting the parallel trends assumption. (Panel a) No usual source of care. (Panel b) Trouble finding a provider. MA, Medicare Advantage; TM, traditional Medicare.

The proportion of adults without a usual source of care decreased following enrollment in MA (13.5% pre-enrollment and 9.9% post-enrollment), with similar patterns among those enrolling in TM (13.9% pre-enrollment and 11.2% post-enrollment). However, there was no differential change between these groups in the post-enrollment relative to the pre-enrollment period (Figure 1a). In contrast, the proportion of adults with trouble accessing medical care stayed relatively stable in both MA (4.0% pre-enrollment and 4.8% post-enrollment) and TM (3.6% pre-enrollment and 4.1% post-enrollment), though again there was no significant differential change (Figure 1b).

### Affordability of health care

**Figure 2:**
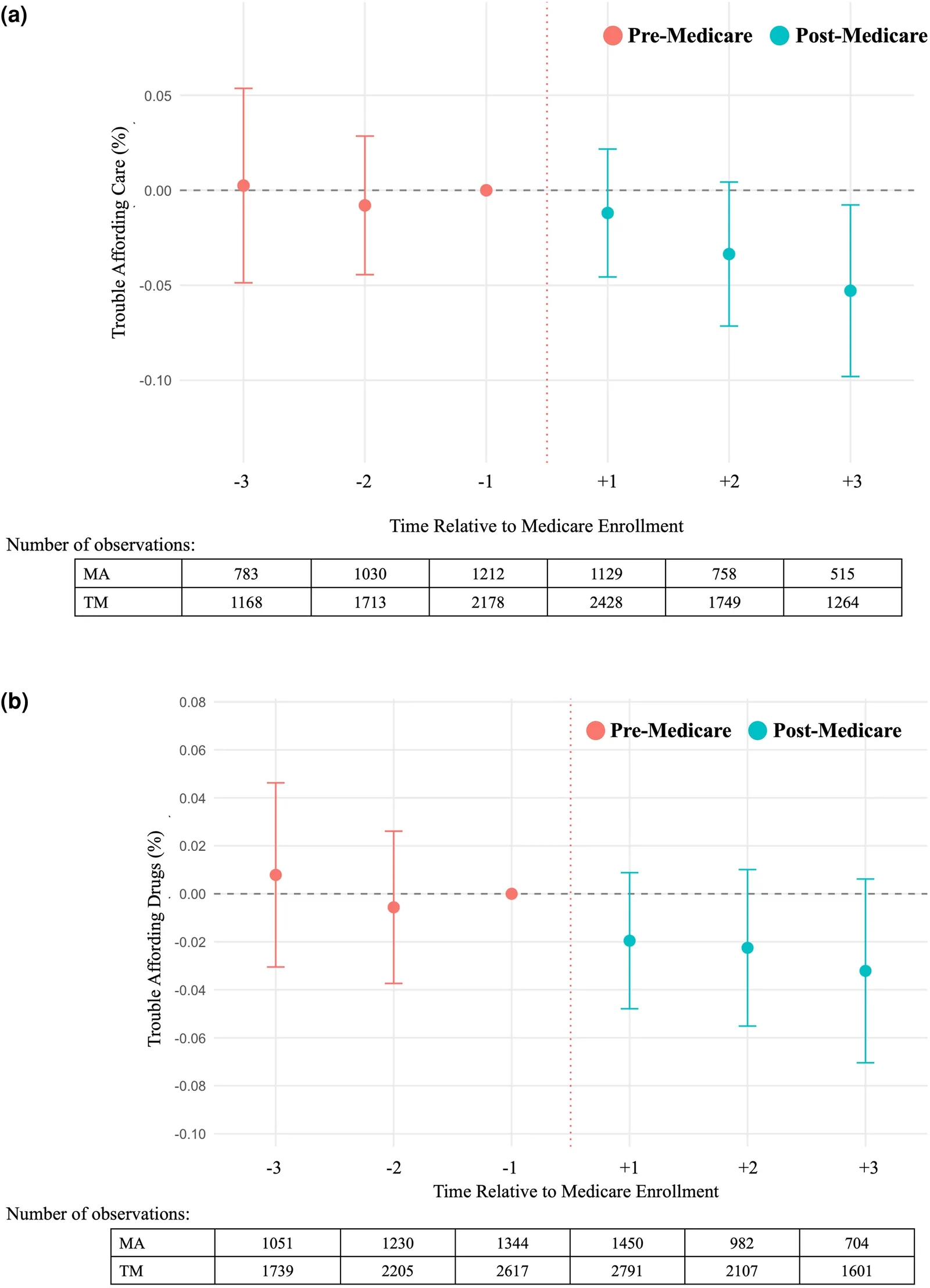
Differential change in the financial burden of care before and after Medicare enrollment. The figures describe the differential change in outcomes of interest by survey cycle before and after Medicare enrollment (vertical dashed line). Participants in the Health and Retirement Study are interviewed every two years; therefore, survey cycle +3 is approximately 6 years after Medicare enrollment. The red dots describe differential change in outcomes before Medicare enrollment, and the blue dots describe differential change in outcomes after Medicare enrollment. The whisker is 95% confidence interval. Each outcome represents the average over a 2-year period. The *P*-values for interaction in the pre-period were 0.94 (trouble affording care) and 0.54 (trouble affording drugs), supporting the parallel trends assumption. (Panel a) Trouble affording medical care. (Panel b) Trouble affording prescription drugs. MA, Medicare Advantage; TM, traditional Medicare.

The proportion of adults who skipped medical care due to costs decreased among both MA (10.7% pre-enrollment and 5.9% post-enrollment) and TM enrollees (8.5% and 4.4% post-enrollment); however, this change was more modest in the TM population. The decrease in deferred medical care became more pronounced among MA compared with TM enrollees over time, with significant differences emerging in the third post-enrollment survey cycle (difference-in-differences [DiD] estimate, −5.3pp [95% CI, −9.7pp to −0.9pp]) (Figure 2a). Fewer MA and TM enrollees also reported trouble affording prescription medications following Medicare enrollment, although the differential change between groups did not quite meet statistical significance (DiD estimate, −3.2pp [95% CI, −6.8pp to 0.4pp] in survey cycle +3) (Figure 2b).

### Satisfaction with health care

**Figure 3:**
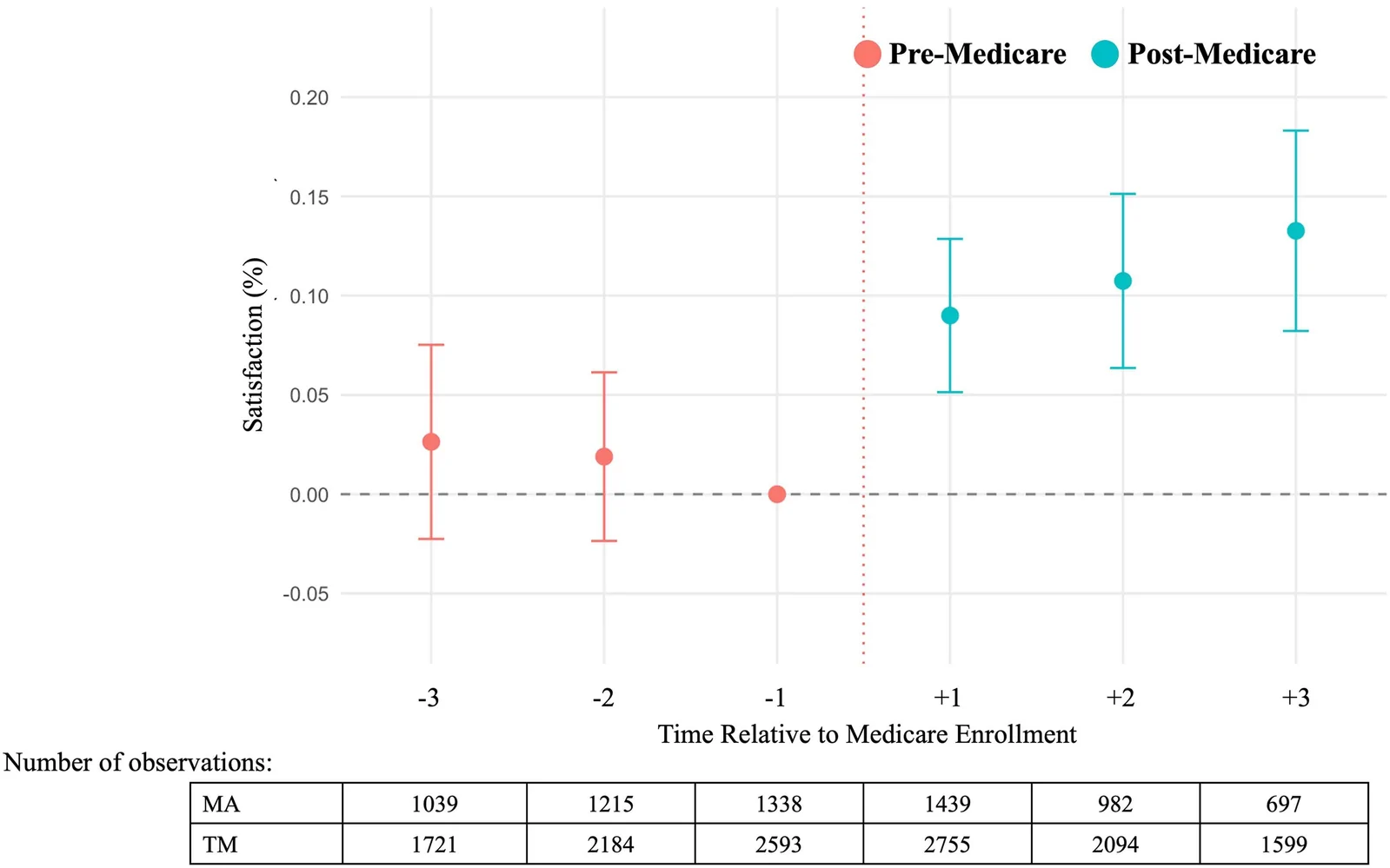
Differential change in satisfaction with care before and after enrollment in Medicare. The figures describe the differential change in outcomes of interest by survey cycle before and after Medicare enrollment (vertical dashed line). Participants in the Health and Retirement Study are interviewed every 2 years; therefore, survey cycle +3 is approximately 6 years after Medicare enrollment. The red dots describe differential change in outcomes before Medicare enrollment, and the blue dots describe differential change in outcomes after Medicare enrollment. The whisker is 95% confidence interval. Each outcome represents the average over a 2-year period. The *P*-value for interaction in the pre-period was 0.19, supporting the parallel trends assumption. MA, Medicare Advantage; TM, traditional Medicare.

The percentage of adults satisfied with their health care increased following enrollment in Medicare (MA: 66.3% pre-enrollment and 84.7% post-enrollment; TM: 69.1% pre-enrollment and 83.2% post-enrollment). This increase in satisfaction was larger among MA enrollees and grew over time (DiD estimate, +9.0pp [95% CI, 5.3pp to 12.9pp] in survey cycle +1 and +13.3pp [95% CI, 7.9pp to 18.6pp] in survey cycle +3) (Figure 3). Pre-enrollment trends were parallel for all analyses based on both visual inspection of the event study plots as well as statistical testing.

In sensitivity analyses, estimates were consistent in direction and magnitude across unadjusted, covariate-adjusted, and IPW specifications (Appendix Table S1). In secondary analyses stratified by baseline insurance type, findings for access to care were consistent with the primary analysis and no significant differential changes were observed across subgroups. The affordability advantage among all MA enrollees observed in the primary analysis was attenuated in the privately insured subgroup, while the satisfaction advantage was broadly consistent across groups but was largest in magnitude among previously uninsured adults. Results for the Medicaid subgroup should be interpreted cautiously given small sample sizes and evidence of non-parallel pre-enrollment trends for one of the access outcomes (no usual source of care) (Appendix Table S2).

## Discussion

In this national, longitudinal analysis of adults enrolling in Medicare at age 65, we found that MA enrollment was not associated with meaningful differences in health care access. However, new MA enrollees experienced greater improvements in affordability of care compared with their TM counterparts and were also more satisfied with their health care, and these differences widened over the follow-up period.

The rapid growth of MA and the substantial federal investment in the program have heightened the urgency to understand how well these private plans serve beneficiaries.^16-18^ We found that beneficiaries reported excellent access to care following enrollment in both Medicare programs. More than 90% of beneficiaries had a usual source of care, and fewer than 5% reported barriers to obtaining needed services after Medicare enrollment. Given persistent concerns that MA network restrictions and prior authorization requirements may limit beneficiaries' ability to obtain needed care, our findings may offer some reassurance. However, our study captures average effects among all newly enrolled beneficiaries, and it is possible that these utilization management strategies create more meaningful barriers for individuals with complex or specialized care needs, or later in life when health needs are greater.

One feature of MA that is particularly attractive to beneficiaries is the presence of annual limits on cost-sharing for Medicare-covered services, a protection not available in TM.^19^ Interestingly, despite this, much of the existing literature has not demonstrated a clear affordability advantage for MA, and in several studies, TM beneficiaries report fewer cost-related barriers to care and better protection from medical expenses.^20-23^ Our findings contrast with this prior work and suggest more favorable financial outcomes among adults entering MA at age eligibility. Several explanations may account for why our findings differ from prior work. In contrast to cross-sectional studies, our design may better control for differences in who selects MA—including situations in which beneficiaries change coverage in response to expensive health shocks—and may better account for baseline demographic differences—particularly the higher prevalence of low-income adults in MA—by observing the same individual before and after enrollment. Alternatively, our cohort consists of relatively younger beneficiaries who are early in their Medicare experience. It is possible that later in life, when health needs and spending increase, the cost-sharing structure of MA could become less favorable and offset the earlier gains we observed—an effect we were not able to assess due to limited follow-up.

Patient experience is a central dimension of Medicare, and satisfaction with care is increasingly used to evaluate plan performance.^24^ We found that satisfaction with care was high among Medicare beneficiaries overall, exceeding 80%. Importantly, satisfaction increased more substantially among individuals newly enrolling in MA compared with those entering TM, and this difference widened over time, culminating in a 13-percentage point gap in satisfaction with the quality, cost, and convenience of care 6 years after enrollment. In secondary analyses stratified by baseline insurance type, this advantage was broadly consistent across groups and was largest among adults who were previously uninsured. Taken together, these findings point to early and sustained differences in beneficiaries' experience of care within MA and underscore the importance of understanding the specific plan features—such as care coordination, supplemental benefits, and cost-sharing structures—that may contribute to higher satisfaction.

Our study provides new evidence on how MA shapes beneficiaries' care and experiences with the health system during their transition into Medicare, a critical period when beneficiaries make initial coverage decisions and early patterns of care may be established. Importantly, by using longitudinal data and observing the same individual before and after Medicare enrollment, we were able to mitigate concerns about nonrandom plan selection and time-invariant differences across beneficiaries that frequently complicate cross-sectional comparisons of MA and TM. We were also able to follow beneficiaries for up to 6 years following enrollment in MA, which allowed us to examine how the effects of managed care evolve over time. Finally, because our analyses relied on survey data prior to Medicare enrollment for risk adjustment, our results should not be influenced by differential coding or diagnostic intensity between groups, and we were also able to examine important patient-centered outcomes not available in administrative datasets.

### Limitations

This study has several limitations. First, all outcomes were based on self-reported survey measures collected every 2 years, which may be subject to recall bias or differential reporting across MA and TM. Second, although our event-study difference-in-differences design helps mitigate confounding from health-based plan sorting at age 65, the causal comparison between MA and TM relies on the assumption—supported by our doubly robust estimator—that plan selection is unrelated to potential outcomes conditional on observed characteristics. This assumption could be violated by unmeasured factors such as systematic differences in the generosity of prior insurance coverage or heterogeneity in local MA plan availability. Third, we were unable to account for heterogeneity across MA plans—including network breadth, prior authorization practices, supplemental benefits, or Star Ratings—or for beneficiaries who switched between MA plans after enrollment. Finally, differential attrition from the HRS over time (eg, if MA beneficiaries with worse experiences or higher costs were more likely to drop out of the survey) could contribute to the widening differences we observed during follow-up and cannot be fully ruled out.

## Conclusion

In this national, longitudinal study of adults entering Medicare, we found that enrollment in MA was associated with greater improvements in the affordability of care and higher satisfaction compared with enrollment in TM, without meaningful differences in health care access.

## Supplementary Material

qxag202_Supplementary_Data
