# Supplementary material for: Health care access, affordability, and satisfaction following enrollment in Medicare Advantage
Source: Health Aff Sch. 2026 Aug 11;4(8):qxag202. doi: 10.1093/haschl/qxag202 (PMC13492085; doi:10.1093/haschl/qxag202)
Supplement: Supplementary file 1 [file qxag202_supplementary_data.zip › Supplementary Material.docx]

**Supplementary Material: Health Care Access, Financial Burden and Satisfaction Following Enrollment in Medicare Advantage**

1. **Figure 1. Flow Diagram**
2. **Table 1. Difference-in-differences Estimates Across Model Specifications**
3. **Table 2. Difference-in-differences Estimates Stratified by Baseline Insurance Type**

**Figure 1. Flow Diagram**

Total HRS participants (2010-2022):
**30,894**

Total Study Population:

**4,247**

**EXCLUDED, N = 26,647**

1. Responded to fewer than 2 cycles: 6,996
2. Enrolled in Medicare prior to age 65: 1,528
3. Earliest available Interview at age >65: 9,734
4. Oldest available age <65: 7,130
5. Non-eligible combo (oldest > 64 and youngest <65): 437
6. Missing insurance: 470
7. Transition to Medicare outside of 65 to 67 years of age: 352

**Table 1.** **Difference-in-Differences Estimates Across Model Specifications**

| **Survey Cycle** | **Unadjusted Estimates** | **Doubly Robust Estimates** | **IPW Estimates** | **Adjusted Regression Estimates** |
| --- | --- | --- | --- | --- |
| No Usual Source of Care | | | | |
| -3 | -0.0159  (-0.0648 to 0.0330) | -0.0159  (-0.0603 to 0.0286) | -0.0158  (-0.0629 to 0.0313) | -0.0164  (-0.0636 to 0.0309) |
| -2 | 0.0208  (-0.0232 to 0.0649) | 0.0222  (-0.0140 to 0.0585) | 0.0234  (-0.0174 to 0.0643) | 0.0238  (-0.0175 to 0.0651) |
| -1 | [Ref] | [Ref] | [Ref] | [Ref] |
| +1 | -0.0199  (-0.0585 to 0.0187) | -0.0214  (-0.0541 to 0.0112) | -0.0206  (-0.0548 to 0.0137) | -0.0204  (-0.0555 to 0.0147) |
| +2 | -0.0166  (-0.0588 to 0.0256) | -0.0156  (-0.0546 to 0.0234) | -0.0166  (-0.0589 to 0.0257) | -0.0166  (-0.0593 to 0.0260) |
| +3 | -0.0323  (-0.0843 to 0.0197) | -0.0359  (-0.0819 to 0.0101) | -0.0341  (-0.0857 to 0.0175) | -0.0323  (-0.0830 to 0.0184) |
| No Trouble Finding a Provider | | | | |
| -3 | -0.0147  (-0.0419 to 0.0125) | -0.0184  (-0.0474 to 0.0106) | -0.0172  (-0.0472 to 0.0127) | -0.0170  (-0.0468 to 0.0127) |
| -2 | -0.0130  (-0.0360 to 0.0099) | -0.0146  (-0.0361 to 0.0069) | -0.0147  (-0.0378 to 0.0084) | -0.0145  (-0.0376 to 0.0086) |
| -1 | [Ref] | [Ref] | [Ref] | [Ref] |
| +1 | -0.0063  (-0.0289 to 0.0164) | -0.0045  (-0.0249 to 0.0159) | -0.0050  (-0.0264 to 0.0164) | -0.0050  (-0.0261 to 0.0162) |
| +2 | -0.0139  (-0.0400 to 0.0122) | -0.0120  (-0.0378 to 0.0138) | -0.0131  (-0.0416 to 0.0153) | -0.0127  (-0.0407 to 0.0153) |
| +3 | -0.0240  (-0.0555 to 0.0074) | -0.0175  (-0.0499 to 0.0148) | -0.0193  (-0.0539 to 0.0152) | -0.0185  (-0.0532 to 0.0163) |
| Trouble affording medical care | | | | |
| -3 | 0.0115  (-0.0404 to 0.0634) | 0.0025  (-0.0464 to 0.0513) | 0.0028  (-0.0511 to 0.0566) | 0.0038  (-0.0504 to 0.0580) |
| -2 | -0.0064  (-0.0456 to 0.0327) | -0.0079  (-0.0463 to 0.0304) | -0.0090  (-0.0511 to 0.0331) | -0.0084  (-0.0502 to 0.0333) |
| -1 | [Ref] | [Ref] | [Ref] | [Ref] |
| +1 | -0.0165  (-0.0482 to 0.0152) | -0.0119  (-0.0420 to 0.0181) | -0.0131  (-0.0472 to 0.0210) | -0.0132  (-0.0473 to 0.0210) |
| +2 | -0.0401  (-0.0781 to -0.002) | -0.0336  (-0.0687 to 0.0016) | -0.0360  (-0.0761 to 0.0041) | -0.0359  (-0.0770 to 0.0051) |
| +3 | -0.0608  (-0.1076 to -0.014) | -0.0529  (-0.0973 to -0.009) | -0.0545  (-0.1024 to -0.0066) | -0.0542  (-0.1025 to -0.0059) |
| Trouble affording prescription drugs | | | | |
| -3 | 0.0120  (-0.0256 to 0.0496) | 0.0079  (-0.0287 to 0.0445) | 0.0093  (-0.0279 to 0.0466) | 0.0095  (-0.0282 to 0.0472) |
| -2 | -0.0029  (-0.0368 to 0.0310) | -0.0056 (-0.0362 to 0.0250) | -0.0055  (-0.0379 to 0.0268) | -0.0052  (-0.0387 to 0.0284) |
| -1 | [Ref] | [Ref] | [Ref] | [Ref] |
| +1 | -0.0187  (-0.0477 to 0.0103) | -0.0195  (-0.0468 to 0.0077) | -0.0195  (-0.0499 to 0.0110) | -0.0195  (-0.0501 to 0.0111) |
| +2 | -0.0289  (-0.0620 to 0.0042) | -0.0225  (-0.0525 to 0.0075) | -0.0254  (-0.0585 to 0.0077) | -0.0253  (-0.0583 to 0.0076) |
| +3 | -0.0391  (-0.078 to -0.0008) | -0.0321  (-0.0683 to 0.0040) | -0.0341  (-0.0748 to 0.0066) | -0.0340  (-0.0748 to 0.0067) |
| Satisfaction with care | | | | |
| -3 | 0.0200  (-0.0313 to 0.0712) | 0.0264  (-0.0273 to 0.0800) | 0.0282  (-0.0282 to 0.0846) | 0.0239  (-0.0321 to 0.0798) |
| -2 | 0.0191  (-0.0282 to 0.0665) | 0.0189  (-0.0230 to 0.0609) | 0.0206  (-0.0247 to 0.0660) | 0.0190  (-0.0267 to 0.0646) |
| -1 | [Ref] | [Ref] | [Ref] | [Ref] |
| +1 | 0.0877  (0.0474 to 0.1279) | 0.0900  (0.0532 to 0.1268) | 0.0884  (0.0487 to 0.1280) | 0.0895  (0.0502 to 0.1287) |
| +2 | 0.1106 (0.0594 to 0.1617) | 0.1074 (0.0643 to 0.1505) | 0.1060 (0.0614 to 0.1507) | 0.1091  (0.0636 to 0.1545) |
| +3 | 0.1371  (0.0839 to 0.1903) | 0.1327  (0.0793 to 0.1860) | 0.1292  (0.0748 to 0.1837) | 0.1337  (0.0785 to 0.1888) |

Estimates reflect the difference-in-differences (DiD) in outcomes between adults newly enrolling in Medicare Advantage versus Traditional Medicare, relative to the survey cycle immediately preceding Medicare enrollment (reference). Values are presented as DiD estimates (95% confidence intervals). Unadjusted estimates reflect the raw DiD without covariate adjustment or propensity score weighting. Doubly robust estimates — used in the primary analysis — combine outcome regression and inverse probability weighting (IPW) and are consistent if either the outcome model or the propensity score model is correctly specified. IPW-only estimates apply propensity score weighting without outcome regression adjustment. Adjusted regression estimates apply covariate adjustment without propensity score weighting.

**Table 2.** **Difference-in-differences Estimates Stratified by Baseline Insurance Type**

| **Survey Cycle** | **Overall Estimates** | **Privately Insured** | **Medicaid** | **Uninsured** |
| --- | --- | --- | --- | --- |
| No Usual Source of Care | | | | |
| -3 | -0.016  (-0.060 to 0.029) | -0.027  (-0.074 to 0.020) | 0.181  (0.009 to 0.354) | -0.042  (-0.183 to 0.100) |
| -2 | 0.0222  (-0.014 to 0.059) | 0.015  (-0.024 to 0.054) | 0.101  (-0.021 to 0.223) | 0.027  (-0.106 to 0.161) |
| -1 | [Ref] | [Ref] | [Ref] | [Ref] |
| +1 | -0.0214  (-0.0541 to 0.0112) | -0.005  (-0.037 to 0.028) | 0.067  (-0.048 to 0.183) | -0.081  (-0.218 to 0.056) |
| +2 | -0.0156  (-0.0546 to 0.0234) | 0.001  (-0.038 to 0.040) | 0.136  (-0.074 to 0.345) | -0.060  (-0.230 to 0.110) |
| +3 | -0.0359  (-0.0819 to 0.0101) | -0.017  (-0.066 to 0.032) | 0.247  (-0.016 to 0.510) | -0.231  (-0.530 to 0.068) |
| No Trouble Finding a Provider | | | | |
| -3 | -0.018  (-0.0474 to 0.0106) | -0.022  (-0.050 to 0.005) | -0.078  (-0.290 to 0.134) | -0.008  (-0.073 to 0.056) |
| -2 | -0.015  (-0.036 to 0.007) | -0.011  (-0.035 to 0.013) | -0.070  (-0.193 to 0.053) | -0.011  (-0.080 to 0.058) |
| -1 | [Ref] | [Ref] | [Ref] | [Ref] |
| +1 | -0.005  (-0.025 to 0.016) | -0.003  -0.026 to 0.020) | -0.043  (-0.164 to 0.078) | -0.015  (-0.078 to 0.048) |
| +2 | -0.012  (-0.038 to 0.014) | -0.012  (-0.039 to 0.016) | 0.015  (-0.190 to 0.220) | -0.020  (-0.133 to 0.094) |
| +3 | -0.018  (-0.050 to 0.015) | -0.018  (-0.052 to 0.016) | -0.023  (-0.314 to 0.269) | -0.030  (-0.159 to 0.098) |
| Trouble affording medical care | | | | |
| -3 | 0.003  (-0.046 to 0.051) | 0.015  (-0.037 to 0.066) | -0.052  (-0.351 to 0.246) | -0.067  (-0.192 to 0.058) |
| -2 | -0.008  (-0.046 to 0.030) | -0.015  (-0.051 to 0.021) | -0.058  (-0.258 to 0.141) | -0.015  (-0.146 to 0.117) |
| -1 | [Ref] | [Ref] | [Ref] | [Ref] |
| +1 | -0.012  (-0.042 to 0.018) | -0.009  (-0.039 to 0.022) | 0.012  (-0.162 to 0.186) | 0.039  (-0.078 to 0.157) |
| +2 | -0.034  (-0.069 to 0.002) | -0.014  (-0.049 to 0.020) | -0.016  (-0.295 to 0.263) | -0.042  (-0.185 to 0.101) |
| +3 | -0.053  (-0.097 to -0.009) | -0.030  (-0.074 to 0.014) | 0.234  (0.045 to 0.423) | N/A |
| Trouble affording prescription drugs | | | | |
| -3 | 0.008  (-0.029 to 0.045) | -0.001  (-0.038 to 0.036) | 0.002  (-0.177 to 0.181) | 0.047  (-0.063 to 0.156) |
| -2 | -0.006  (-0.036 to 0.025) | -0.004  (-0.035 to 0.027) | -0.024  (-0.207 to 0.159) | 0.000  (-0.113 to 0.113) |
| -1 | [Ref] | [Ref] | [Ref] | [Ref] |
| +1 | -0.020  (-0.047 to 0.008) | -0.003  (-0.030 to 0.025) | -0.060  (-0.209 to 0.089) | -0.031  (-0.125 to 0.063) |
| +2 | -0.023  (-0.053 to 0.008) | -0.009  (-0.043 to 0.024) | -0.082  (-0.295 to 0.131) | 0.030  (-0.091 to 0.151) |
| +3 | -0.032  (-0.068 to 0.004) | -0.021  (-0.059 to 0.018) | -0.132  (-0.369 to 0.105) | -0.030  (-0.196 to 0.137) |
| Satisfaction with care | | | | |
| -3 | 0.026  (-0.027 to 0.080) | 0.020  (-0.038 to 0.077) | -0.028  (-0.244 to 0.189) | 0.066  (-0.089 to 0.221) |
| -2 | 0.019  (-0.023 to 0.061) | 0.006  (-0.045 to 0.057) | 0.083  (-0.087 to 0.254) | 0.070  (-0.081 to 0.221) |
| -1 | [Ref] | [Ref] | [Ref] | [Ref] |
| +1 | 0.090  (0.053 to 0.127) | 0.052  (0.004 to 0.100) | 0.129  (-0.039 to 0.298) | 0.254  (0.126 to 0.381) |
| +2 | 0.107  (0.064 to 0.151) | 0.086  (0.035 to 0.136) | 0.163  (-0.108 to 0.434) | 0.212  (0.052 to 0.372) |
| +3 | 0.133  (0.079 to 0.186) | 0.098  (0.037 to 0.158) | 0.098  (-0.284 to 0.481) | 0.146  (-0.055 to 0.347) |
